# Supplementary material for: The Construction and Exploration of a Comprehensive MicroRNA Centered Regulatory Network in Foxtail Millet (Setaria italica L.)
Source: Front Plant Sci. 2022 May 6;13:848474. doi: 10.3389/fpls.2022.848474 (PMC9121102; doi:10.3389/fpls.2022.848474)
Supplement: Supplementary file 4 [file Data_Sheet_4.zip › Sit-miR5568_Seita.2G207900.1_2292_TPlot.pdf]

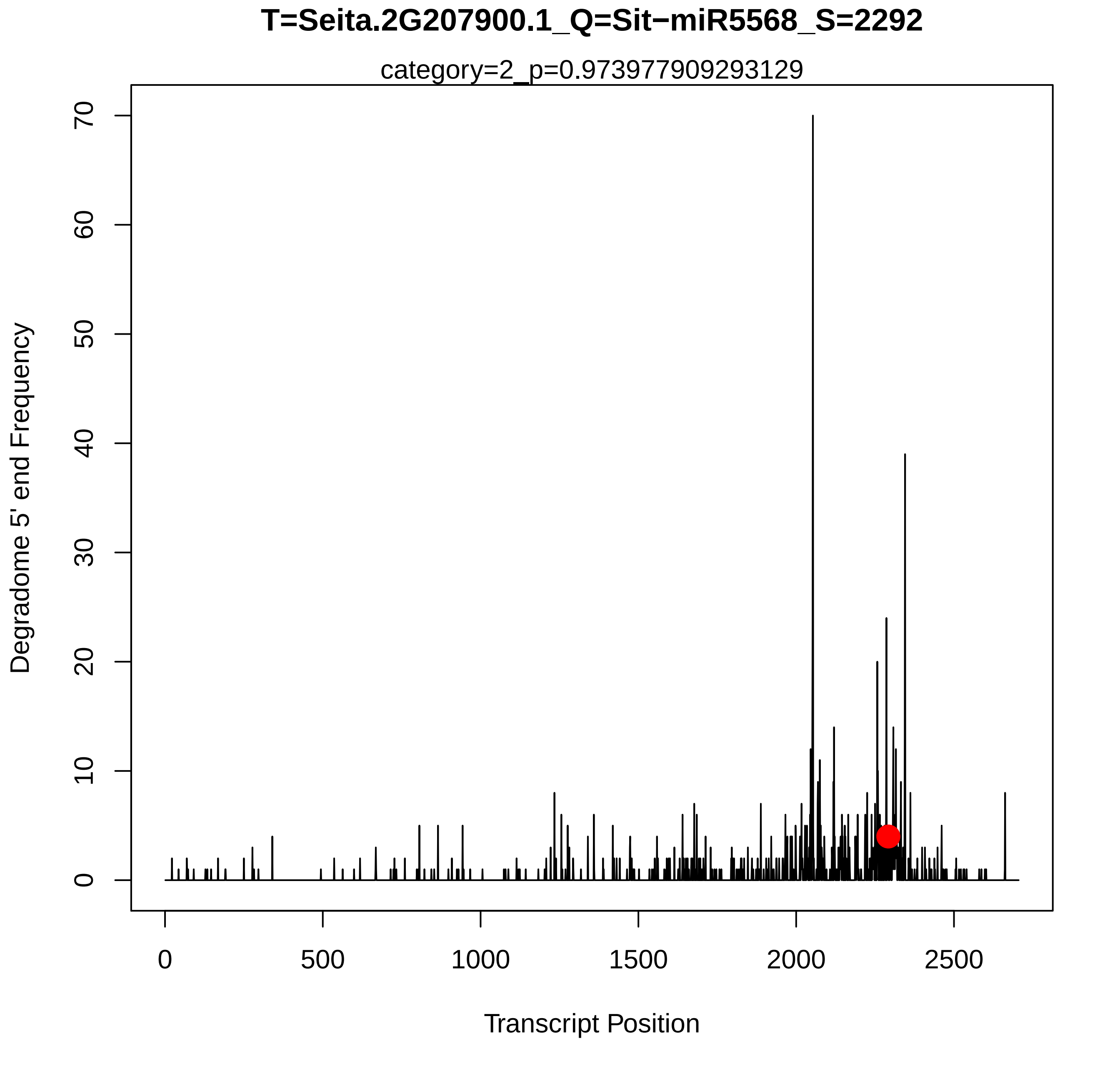
T=Seita.2G207900.1_Q=Sit−miR5568_S=2292
category=2_p=0.973977909293129
Degradome 5' end Frequency
Transcript Position
0
10
20
30
40
50
60
70
0
500
1000
1500
2000
2500
